# Haematopoietic malignancies in Côte d'Or (France): a population based study.

**DOI:** 10.1038/bjc.1986.137

**Published:** 1986-06

**Authors:** P. M. Carli, C. Milan, A. Lange, E. Devilliers, H. Guy, J. Faivre

## Abstract

A registry of haematopoietic malignancies was established on January 1, 1980 in order to accurately determine the incidence and epidemiological features of these diseases in the department of Côte d'Or (population 478,000). Over five years (1980-1984), 704 new cases were recorded. The crude incidence rates were 32.7 per 100,000 for males and 24.9 per 100,000 for females. The corresponding age standardized rates were 26.4 and 16.7. The sex ratio was 1.6:1. In males, chronic lymphocytic leukaemias were the most common haematopoietic malignancies, followed by non Hodgkin's lymphomas, acute leukaemias and multiple myelomas. In females, multiple myelomas and acute leukaemias preceded non Hodgkin's lymphomas and chronic lymphocytic leukaemias. For men and women, the risk of haematopoietic malignancies was higher in urban areas than in rural areas. Compared to population based registries in other countries, incidence rates are among the highest reported and are particularly high for chronic lymphocytic leukaemia.


*Br. J. Cancer* (1986), **53**, 811–815

### Haematopoietic malignancies in Côte d'Or (France): A population based study

P.M. Carli[1], C. Milan[2], A. Lange[1], E. Devilliers[1], H. Guy[3] & J. Faivre[2]

[1]*Registre des Hémopathies Malignes*; [2]*Registre des Tumeurs Digestives*; [3]*Clinique Médicale, Chu Dijon, France*

**Summary** A registry of haematopoietic malignancies was established on January 1, 1980 in order to accurately determine the incidence and epidemiological features of these diseases in the department of Côte d'Or (population 478,000). Over five years (1980–1984), 704 new cases were recorded. The crude incidence rates were 32.7 per 100,000 for males and 24.9 per 100,000 for females. The corresponding age standardized rates were 26.4 and 16.7. The sex ratio was 1.6:1. In males, chronic lymphocytic leukaemias were the most common haematopoietic malignancies, followed by non Hodgkin's lymphomas, acute leukaemias and multiple myelomas. In females, multiple myelomas and acute leukaemias preceeded non Hodgkin's lymphomas and chronic lymphocytic leukaemias. For men and women, the risk of haematopoietic malignancies was higher in urban areas than in rural areas. Compared to population based registries in other countries, incidence rates are among the highest reported and are particularly high for chronic lymphocytic leukaemia.

The only data concerning the incidence of haematopoietic malignancies prior to 1975 in France came from death certificates. However, these certificates are often imprecise or incomplete. Therefore, French mortality data were not included in the last volume of Cancer Incidence in Five Continents (Muir & Waterhouse, 1982). Since 1975, four registries have been established in France (Schraub, 1983). A population based registry specializing in haematopoietic malignancies was created in the department of Côte d'Or in 1980. As population based registries in France are recent, incidence data about haematopoietic malignancies in this country are not yet well known; the purpose of this study is to report the incidence and the characteristics of haematopoietic malignancies in the department of Côte d'Or.

### Materials and methods

The study included all patients residing in the department of Côte d'Or in whom a haematopoietic malignancy was diagnosed for the first time between January 1, 1980 and December 31, 1984. The population of Côte d'Or consisted of 234,900 males and 243,100 females (Schmitt, 1981). Information was collected from public and private biological laboratories, local and private hospital departments, and general practitioners; death certificates were also collected. Patients were registered in three indexes (by name, by date of birth and by nature of haemopathy) to eliminate duplication.

This registration took place under excellent conditions; biological information exists for all cases, clinical information in 98% of the cases, and there was an average of three notifications per case. The morbidity/mortality ratio was 1.9.

The registration included all haematopoietic malignancies and dysmyelopoietic syndromes: acute leukaemias, lymphoid and myeloid proliferative disorders, Hodgkin's and non Hodgkin's lymphomas. Acute leukaemias and dysmyelopoietic syndromes were classified according to the FAB co-operative group (Bennet *et al.*, 1976). The 8th International Classification of Diseases was also adopted (World Health Organization, 1967) to permit comparison with other countries (Waterhouse *et al.*, 1982) (ICD codes 200 to 209 inclusive).

Population data used in calculating incidence rates were based on estimates of the Côte d'Or population by age and sex, calculated annually by the Institut National de la Statistique et des Etudes Economiques (INSEE) by extrapolation from the 1975 census (Schmitt, 1981). For the purpose of regional comparison, rates have been standardized by the direct method using the world standard population (Segi & Kurihara, 1969).

### Results

### *Incidence by site and sex*

There were 704 newly diagnosed cases of haematopoietic malignancies registered from 1980 to 1984,

Correspondence: P.M. Carli
Received 24 October 1985; and in revised form, 12 February 1986

© The Macmillan Press Ltd., 1986

among Côte d'Or residents. The rates standardized according to the world standard population were 26.4 for males and 16.7 for females. The sex ratio was 1.6:1. Incidence rates by sex and nature of haemopathy are given in Table I. In males, chronic lymphocytic leukaemias rank first (20% of the cases) before non Hodgkin's lymphomas (16%), acute leukaemias (15%), multiple myelomas (12%), myeloproliferative diseases (14%), Hodgkin's diseases (8%), dysmyelopoietic syndromes (8%) and other lymphoproliferative diseases (7%). In females, multiple myelomas rank first (17% of the cases) with acute leukaemias (17%); they precede non Hodgkin's lymphomas (16%), chronic lymphocytic leukaemias (16%), dysmyelopoietic syndromes (11%), myeloproliferative diseases (10%) and other lymphoproliferative diseases (5%).

*Incidence by age*

For the most frequent haemopathies, age and sex specific incidence rates are given in Figure 1. In acute leukaemias, incidence rates in males and females were comparable. In chronic lymphocytic leukaemias, incidence rates were low before 50 years of age and rose in the older age groups, with a male predominance. In Hodgkin's lymphoma there were no cases before 5 years of age, and incidence was higher between 20 and 55 years old. In non Hodgkin's lymphoma, the incidence increased in all age groups over 55 years.

The mean ages of the patients at time of diagnosis by type of haematopoietic malignancy are given in Table II. There were no significant differences except for chronic lymphocytic leukaemias: females patients were older than males ($P < 0.05$).

*Urban–rural differences*

There were no significant variations in incidence for the different types of haematopoietic malignancy, but there was a trend towards a higher incidence in urban than in rural areas for all types except acute leukaemias in females. When rates were added, the risk of haematopoietic malignancies was significantly higher in urban than in rural areas. In males, the age standardized rates were respectively 30.9/100,000 and 20.4/100,000 ($P < 0.01$). In females, they were 18.7/100,000 and 13.9/100,000 ($P < 0.05$).

*Classification of acute leukaemias and dysmyelopoietic syndromes*

They were classified according to FAB classification. Out of 112 cases of acute leukaemias, 35 were acute lymphoid leukaemias: there were 21 L1 (59%), 11 L2 (32%), 3 L3 (9%); and 77 acute myeloid leukaemias: there were 14 M1 (18%), 21 M2 (27%), 5 M3 (7%), 14 M4 (18%), 12 M5 (16%), 3 M6 (4%), 8 rare type (10%). Out of 69 dysmyelopoietic syndromes, there were 16 sidero-

**Table I** Incidence of haematopoietic malignancies by sex

| *ICD* | *Haemopathy* | *Number of cases* | | *Crude incidence rates* | | *Age standardized rate* | | |
|---|---|---|---|---|---|---|---|---|
| | | *M* | *F* | *M* | *F* | *M* | *F* | *Sex ratio* |
| 200 | Non Hodgkin's lymphomas | 64 | 48 | 5.4 | 3.9 | 4.5 | 2.5 | 1.8 |
| 201 | Hodgkin's | 32 | 26 | 2.7 | 2.1 | 2.4 | 2.1 | 1.1 |
| 204–1 | Chronic lymphocytic leukaemias | 80 | 48 | 6.7 | 3.8 | 5.2 | 1.8 | 2.9 |
| 204–0 | Acute lymphoid leukaemia | 20 | 15 | 1.7 | 1.2 | 1.8 | 1.4 | 1.3 |
| 205–0 206–0 | Acute myeloid and monocytic leukaemia | 39 | 38 | 3.3 | 3.1 | 2.5 | 2.3 | 1.1 |
| 203 | Multiple myelomas | 45 | 52 | 3.9 | 4.4 | 2.9 | 2.7 | 1.1 |
| | Waldenström disease | 14 | 3 | 1.2 | 0.2 | 0.8 | 0.2 | 4.0 |
| 204–2 | Other lymphoproliferative diseases | 13 | 7 | 1.1 | 0.7 | 0.9 | 0.5 | 1.8 |
| 205–1 | Chronic myeloid leukaemias | 22 | 4 | 1.9 | 0.3 | 1.7 | 0.2 | 8.5 |
| 208 | Polycythaemia vera | 16 | 10 | 1.3 | 0.8 | 1.0 | 0.7 | 1.4 |
| 209 | Myelofibrosis | 6 | 5 | 0.5 | 0.4 | 0.3 | 0.3 | 1.0 |
| 205–2 | Other myelo-proliferative diseases | 12 | 13 | 1.0 | 1.1 | 0.7 | 0.8 | 0.9 |
| | Dysmyelopoietic syndromes | 34 | 35 | 2.9 | 2.9 | 1.7 | 1.4 | 1.2 |
| | Unspecified type | 2 | 1 | 0.1 | 0.0 | — | — | — |
| | All haematopoietic malignancies | 399 | 305 | 32.7 | 24.9 | 26.4 | 16.7 | |

Not coded in ICD 8

**Figure 1** Age specific incidence rate for the most frequent haematopoietic malignancies. Males (——); females (-----).

**Table II** Mean ages of the patients by type of haematopoietic malignancy

| | *Males* | *Females* | *P* |
|---|---|---|---|
| Hodgkin's disease | 36.5±16.8 | 35.6±19.4 | NS |
| Non Hodgkin's lymphoma | 60.7±14.3 | 64.3±17.6 | NS |
| Chronic lymphocytic leukaemia | 68.2±12.7 | 73.5±13.3 | <0.05 |
| Acute lymphoid leukaemia | 24.0±26.4 | 23.9±24.1 | NS |
| Acute myeloid leukaemia | 63.4±21.4 | 56.5±26.3 | NS |
| Multiple myeloma | 70.8±11.4 | 70.6±10.7 | NS |
| Chronic myeloid leukaemia | 53.4±16.9 | 49.5±21.7 | NS |
| Polycythaemia vera | 66.9±10.8 | 60.3±12.5 | NS |
| Myelofibrosis | 69.8± 7.6 | 71.6±14.9 | NS |
| Dysmyelopoietic syndrome | 71.3±13.7 | 74.6±11.3 | NS |

blastic anaemias (23%), 32 refractory anaemias with excess of blasts (46%), 1 refractory anaemia without excess of blasts (1%), 14 chronic myelomonocytic leukaemias (20%).

### Discussion

One of the major problems faced by cancer registries is the determination of completeness and reliability of the data. Because of the high level of participation of the whole medical profession in the department, a large proportion of newly diagnosed haemopathies was registered. It has been checked that there were no prevalent cases in the registered cases.

In this paper, in order to compare our results with those of other registries (Waterhouse *et al.*, 1982), it was necessary to use the 8th revision (World Health Organization, 1967). Available classification of haematopoietic malignancies involved difficulties: in the 7th revision, it was not even possible to separate acute and chronic leukaemias. In the 8th revision, myelodysplastic syndromes were not included (except chronic monocytic leukaemias). In the 9th revision, they were included but in ICD codes far from those of haematopoietic malignancies (ICD 284/285–0). In the 10th revision, it would be unfortunate not to use the FAB classification concerning acute leukaemias and dys-

**Table III** Age-adjusted incidence rates for haematopoietic malignancies by sex (from Waterhouse *et al.* (1982) variable period between 1972 and 1977)

| | *ICD 8th* | *Lymphosar-coma etc.* | *Hodgkin's disease* | *Other re-ticuloses* | *Multiple myeloma* | *Lymphatic leukaemia* | *Myeloid leukaemia* | *Monocytic leukaemia* | *Other leukaemias* | *Polycythae-mia vera* | *Myelofi-brosis* | *All haemat. malign.* |
|---|---|---|---|---|---|---|---|---|---|---|---|---|
| Switzerland | M | 8.5 | 3.5 | 1.8 | 2.6 | 2.9 | 4.5 | 0.1 | 0.4 | 0.1 | 0.8 | 25.2 |
| (Geneva) | F | 4.5 | 1.3 | 0.9 | 2.0 | 2.5 | 3.1 | 0.2 | 0.2 | 0.2 | 0.3 | 15.2 |
| USA | M | 5.5 | 4.0 | 2.5 | 3.4 | 4.7 | 4.2 | 0.2 | 1.4 | — | — | 25.9 |
| (Connecticut) | F | 4.8 | 2.8 | 1.9 | 2.7 | 2.7 | 2.9 | 0.2 | 0.8 | — | — | 18.8 |
| United Kingdom | M | 3.4 | 3.1 | 2.2 | 2.8 | 3.0 | 2.7 | 0.2 | 0.4 | 0.8 | 0.4 | 19.0 |
| (Oxford) | F | 2.1 | 1.9 | 1.5 | 1.6 | 2.0 | 1.9 | 0.1 | 0.4 | 0.4 | 0.3 | 12.2 |
| Norway | M | 3.4 | 2.6 | 2.1 | 3.7 | 3.1 | 3.1 | 0.1 | 1.1 | — | — | 16.2 |
| | F | 2.5 | 1.6 | 1.4 | 2.4 | 1.8 | 2.5 | 0.1 | 0.7 | — | — | 13.0 |
| Colombia | M | 6.0 | 2.8 | — | 1.8 | 2.4 | 4.2 | 0.2 | 0.5 | 0.0 | 0.0 | 17.9 |
| (Coli) | F | 2.8 | 1.1 | — | 1.1 | 1.7 | 1.6 | 0.2 | 0.7 | 0.0 | 0.0 | 9.2 |
| Japan | M | 2.2 | 0.5 | 1.3 | 1.1 | 0.6 | 2.6 | 0.4 | 0.9 | 0.1 | 0.0 | 9.7 |
| (Myagi) | F | 0.8 | 0.2 | 0.9 | 0.7 | 0.7 | 1.9 | 0.3 | 1.0 | 0.0 | 0.0 | 6.5 |
| France | M | 4.6 | 3.1 | 0.7 | 2.5 | 4.7 | 3.1 | 0.1 | 0.6 | 0.7 | 0.3 | 20.4 |
| (Bas Rhin) | F | 3.1 | 1.8 | 0.2 | 1.2 | 2.5 | 2.4 | 0.2 | 0.3 | 1.0 | 0.1 | 12.8 |
| France | M | 2.4 | 0.8 | 3.7 | 1.6 | 4.5 | 0.5 | 0.0 | 0.0 | 0.0 | 2.1 | 15.6 |
| (Doubs) | F | 2.2 | 3.2 | 1.4 | 0.6 | 3.4 | 0.6 | 0.0 | 0.3 | 0.0 | 0.3 | 12.0 |
| France | M | 4.5 | 2.5 | 0.0 | 2.9 | 8.7 | 4.3 | 1.2 | 1.0 | 1.0 | 0.3 | 26.4 |
| (Côte d'Or) | F | 2.5 | 2.1 | 0.0 | 2.7 | 3.9 | 3.1 | 0.4 | 1.0 | 0.7 | 0.3 | 16.7 |

myelopoietic syndromes, seeing that classification is actually used by most of the haematologists.

One of the most important uses of registered data is to permit comparison with the data of cancer registries in other countries (Table III). For the whole range of haematopoietic malignancies, high rates are reported in the department of Côte d'Or as well as in most West European and North American countries. Incidence rates are in the intermediate range in Norway and South Africa and they are low in Asia (particularly in Japan). They are higher in Côte d'Or than in other population-based French registries. It must be underlined that an unexpected high incidence rate for chronic lymphocytic leukaemias was found. There are two possible explanations for such a special situation. One is that the incidence of chronic lymphocytic leukaemia in Côte d'Or does not differ from that in other places, except that the reporting of the cases is more complete in Côte d'Or. The other is that chronic lymphocytic leukaemia is really more frequent in our department. Case-control studies are necessary to search for environmental and occupational factors. The results could explain this high incidence rate. For multiple myeloma, the rates are in the intermediate range, lower than in countries with a high black population. Hodgkin's and non-Hodgkin's lymphomas are frequent but less than in Geneva (Switzerland), a very high risk region. For the other haematopoietic malignancies. Côte d'Or rates are similar to those reported in most countries. As in most cancer registries, the male/female ratio of incidence rates was about 1:1 for acute leukaemias (as for everywhere in the world), while there was a high male predominance for chronic leukaemias.

Comparison of urban and rural incidence data are difficult because the definitions of urban and rural vary a great deal from one area of the world to another. The definition of urban and rural used in France is based on the size of the population; urban areas include agglomerations of more than 2,000 inhabitants. Although comparisons need to be treated with caution, they show a similar trend; there was a tendency towards a slightly higher urban rate for most of the types of haematopoietic malignancies (significantly higher for whole haemopathies). In Iowa, an increasing incidence of chronic lymphocytic leukaemias from urban to rural areas was noted (Donham *et al.*, 1980). This fact suggests the possibility of an environmental exposure. The purpose of population-based registries is to search for aetiologic factors. They are not yet known except for therapeutic drugs and radiation.

There are no comparable population-based statistics concerning classification of acute leukaemias and myelodysplastic syndromes according to FAB classification. In acute lymphoid leukaemias, the L1 was the predominant type, and in acute myeloid leukaemias the M2 type was more frequent than M1, M4 and M5 types. For dysmyelopoietic syndromes, the refractory anaemias with excess of blasts represent about half of the cases. Comparison with data from Iowa (Dick *et al.*, 1982) shows similar results except for L1 and L2 classes. However, Côte d'Or data include children, and those of Dick *et al.* only adults. These data are interesting as they concern an overall population. They provide clinicians with data for reference and public health authorities with a basis for monitoring cancer patients.

This work was supported by the 'Ligue Nationale contre le Cancer' and the 'Association de Recherche contre le Cancer'. The authors acknowledge the Côte d'Or biologists and practitioners for their participation. The authors thank E. Gauthier for her translation and typing.

### References

BENNET, J.M., CATOVSKY, D., DANIEL, M.T. & 4 others (1976). Proposals for the classification of acute leukaemias. *Br. J. Haematol.*, **33**, 451.

DICK, R.F., ARMITAGE, O.J. & BURNS, C.P. (1982). Diagnostic concurrence in the subclassification of adult acute leukemia using F.A.B. criteria, *Cancer*, **49**, 916.

DONHAM, K.J., BERG, J.W. & SANIN, R.S. (1980). Epidemiologic relationship of the bovine population and human leukemia in Iowa. *Am. J. Epidemiol.* **112**, 80.

MUIR, C.S. & WATERHOUSE, J. (1982). Comparability of the data and reliability of registration. In *Cancer Incidence in Five Continents*, **IV**, (ed) p. 55. IARC Scient. publ., no. 42, Lyon.

SCHMITT, G. (1981). Prudent: modèle de projection démographique. In *Dimensions Economiques de la Bourgogne*, **22**, 29.

SCHRAUB, S., FAIVRE, J., GIGNOUX, M., MENEGOZ, F., ROBILLARD, J. & SCHAEFFER, P. (1983). Cancer registries. *Effective Health Care*, **1**, 205.

SEGI, M. & KURIHARA, M. (1969). *Cancer Mortability of Selected Sites in 24 Countries*, No. 5, 1964–1965. Tohoku University School of Medicine, Sendai, Japan.

WATERHOUSE, J., MUIR, C., SHANMUGARATNAM, K. & POWELL, J. (1982). *Cancer Incidence in Five Continents*, **IV**, IARC Scient. Publ., no. 42, Lyon.

WORLD HEALTH ORGANIZATION (1967). *International Classification of Diseases*, 8th revision, Geneva.


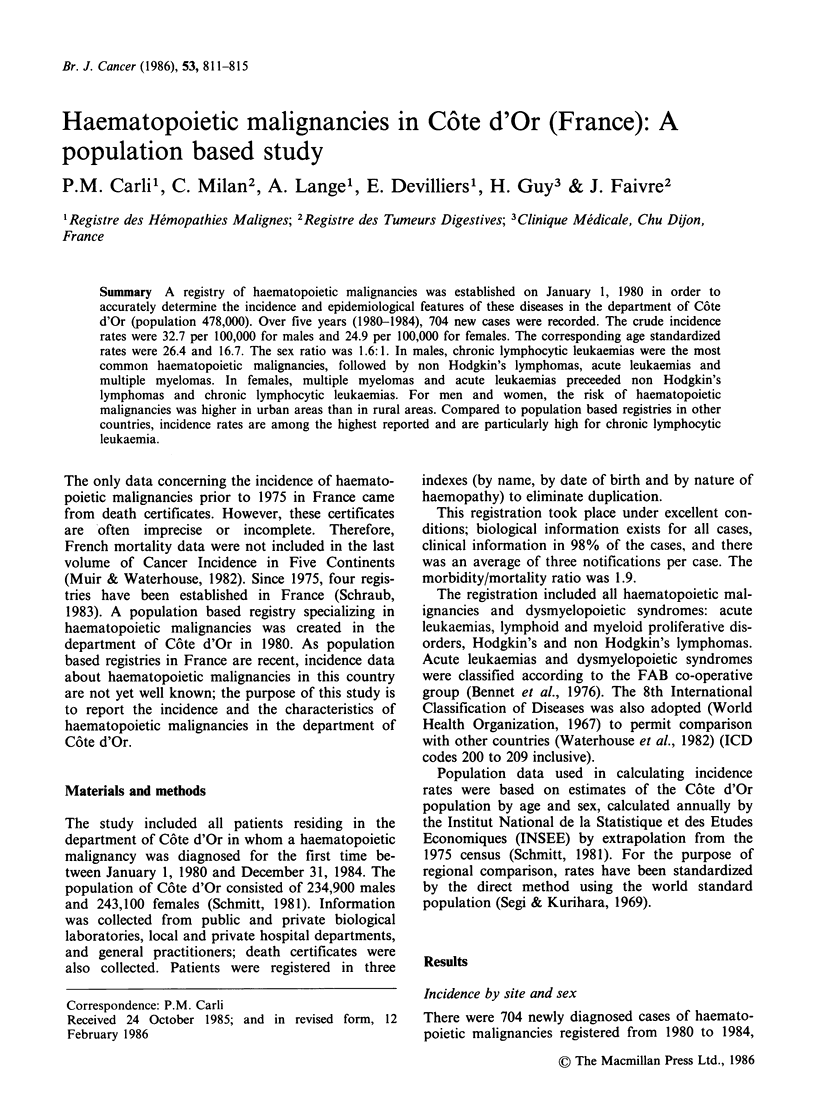

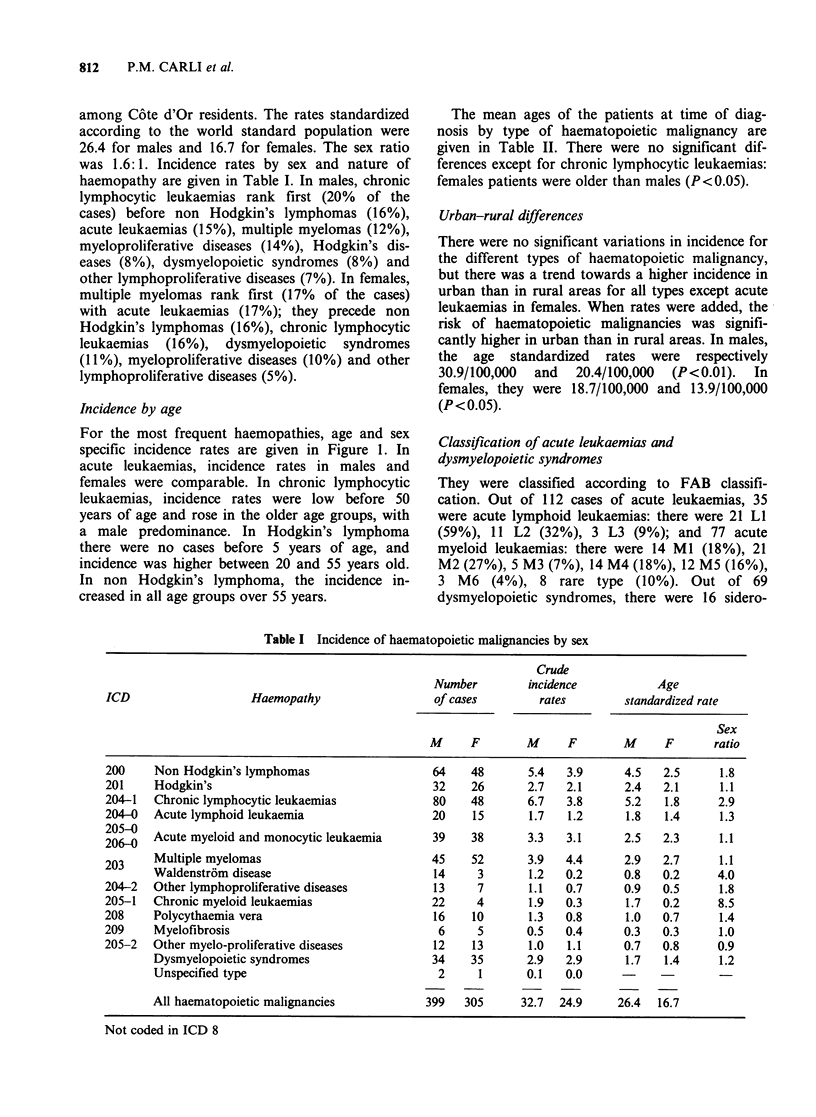

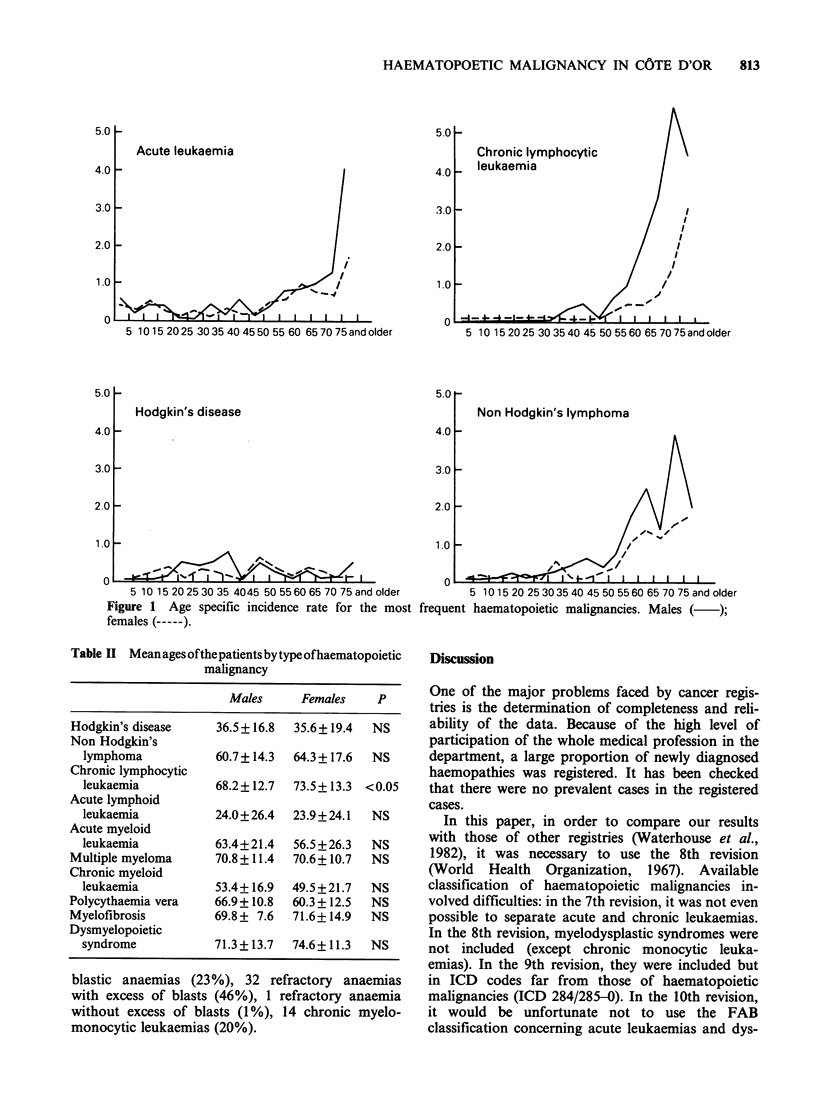

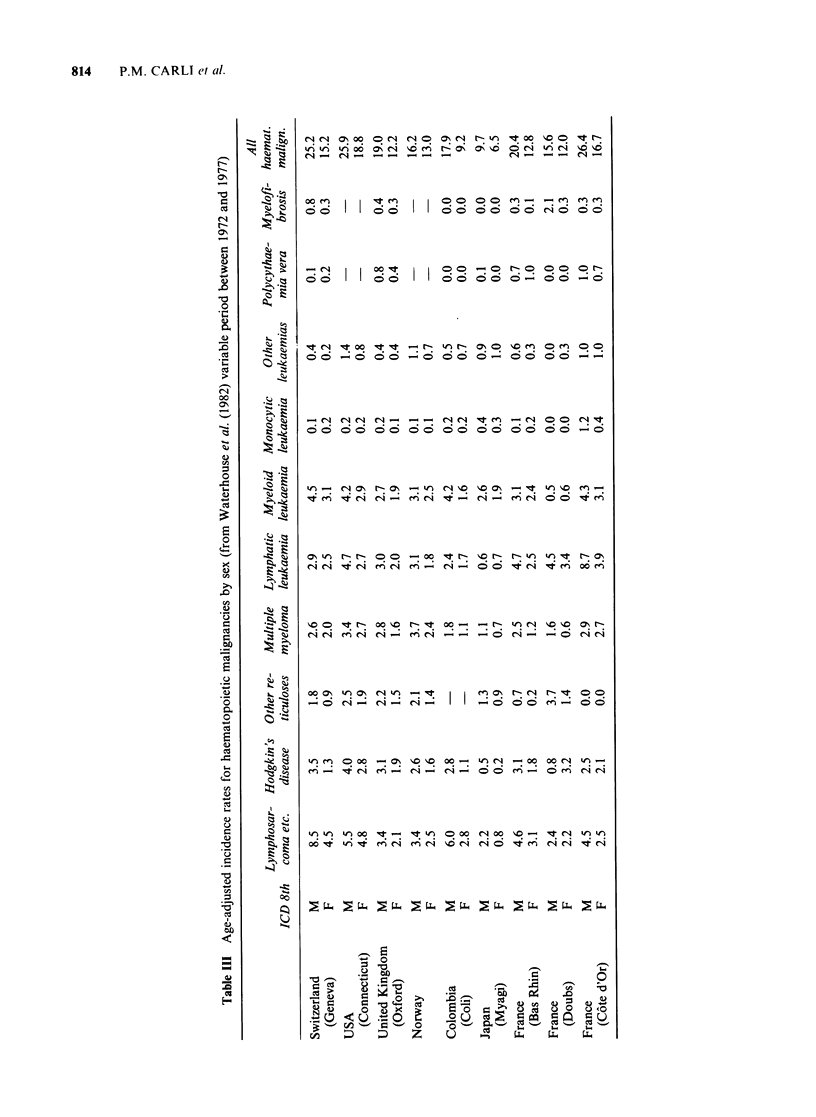

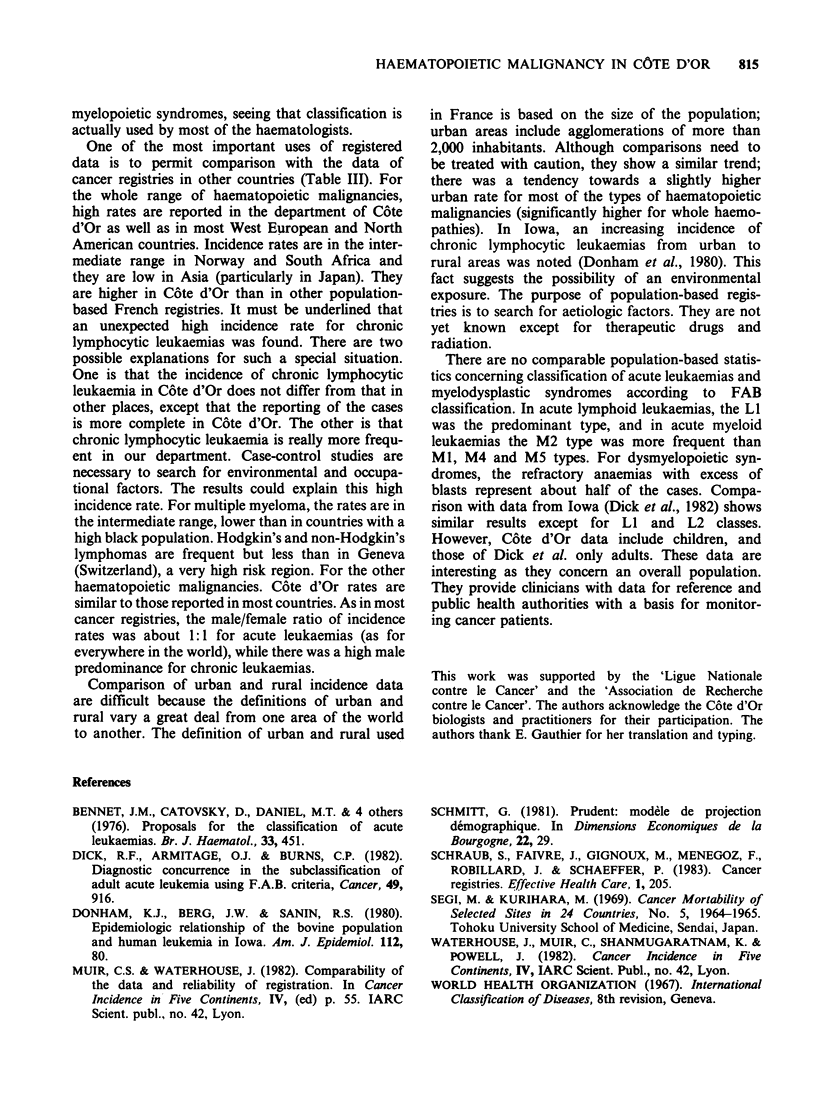


## References

[OCR_00696] Bennett J. M., Catovsky D., Daniel M. T., Flandrin G., Galton D. A., Gralnick H. R., Sultan C. (1976). Proposals for the classification of the acute leukaemias. French-American-British (FAB) co-operative group.. Br J Haematol.

[OCR_00701] Dick F. R., Armitage J. O., Burns C. P. (1982). Diagnostic concurrence in the subclassification of adult acute leukemia using French-American-British criteria.. Cancer.

[OCR_00707] Donham K. J., Berg J. W., Sawin R. S. (1980). Epidemiologic relationships of the bovine population and human leukemia in Iowa.. Am J Epidemiol.

[OCR_00724] Schraub S., Faivre J., Gignoux M., Menegoz F., Robillard J., Schaffer P. (1983). Cancer registries: their interest and practical problems.. Eff Health Care.

